# Maxillary right second molar with two palatal root canals

**DOI:** 10.4103/0972-0707.66720

**Published:** 2010

**Authors:** M B Prashanth, Pradeep Jain, Pallav Patni

**Affiliations:** Department of Endodontics and Conservative Dentistry, Devi Ahilya University, Sri Aurobindo College of Dentistry, Indore, MP- 01, India

**Keywords:** Access opening, maxillary second molar, palatal canals, root canal morphology, variations

## Abstract

We report a clinical case of maxillary right second molar with two palatal root canals. The morphology is atypical because it is characterized by two palatal roots with two canals with widely separated orifices and canals. Modifications to the normal access opening and examination of the pulpal floor for additional canals are stressed.

## INTRODUCTION

The hard tissue repository of the human dental pulp takes on numerous configurations and shapes. A thorough knowledge of tooth morphology, careful interpretation of angled radiographs, proper access preparation and a detailed exploration of the interior of the tooth are essential prerequisites for a successful treatment outcome. Magnification and illumination are aids that must be utilized to achieve this goal.[1]

Most studies in anatomical variations of maxillary molar teeth appear to deal with the first molar. Anatomical variation studies in the second maxillary molars are not so numerous. Fava *et al*,[2] reported the presence of just one canal and one root in the second maxillary molars of the same patient, while Alani AH[3] encountered four roots in the second maxillary molars of the same patient bilaterally. Baratto-Filho *et al*,[4] carried out an *in vitro* study of two maxillary second molars with four canals and two different palatal roots. In the same year, Barbizam *et al*,[5] reported a similar study of a second maxillary molar also with four canals in four distinct roots. It is worth mentioning that Benenati (2004) presented a clinical case of a second maxillary molar with two palatal roots and a groove located at the side of the tooth.

The purpose of the present study is to report a clinical case of maxillary second molar with two separate palatal canals, a rare entity.

## CASE REPORT

**Figure 1:**
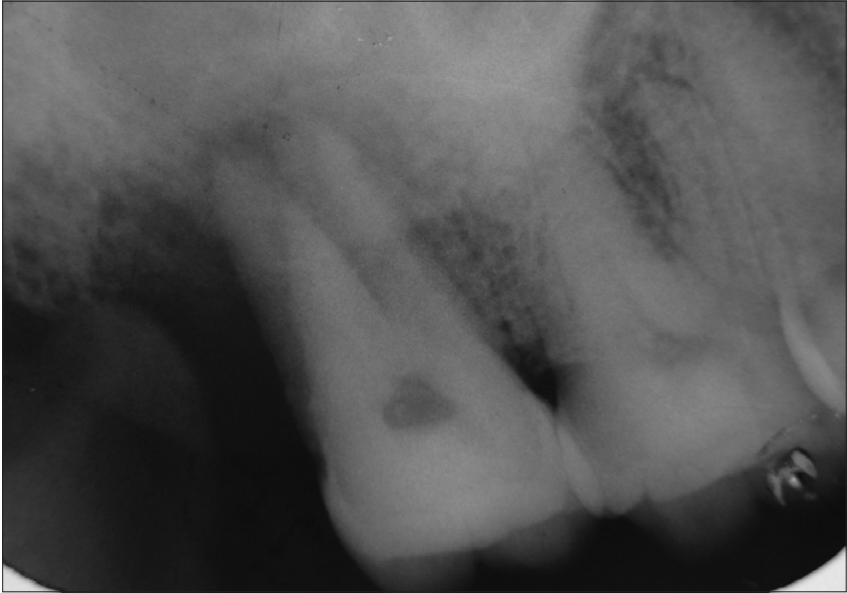
Pre-op radiograph

A 32-year-old male patient reported to the out patient department of conservative dentistry. He presented with a chief complaint of mild pain in the maxillary right posterior area since one month. The medical status was unremarkable. Clinical examinations revealed that the tooth had no caries, was mildly tender to vertical percussion and palpation. Radiographic examination disclosed periapical widening with distal bone loss with grade-I mobility and a bizarre anatomical configuration of the root canals [Figure 1]. The tooth did not respond on vitality test examination (EPT and thermal test)

**Figure 2:**
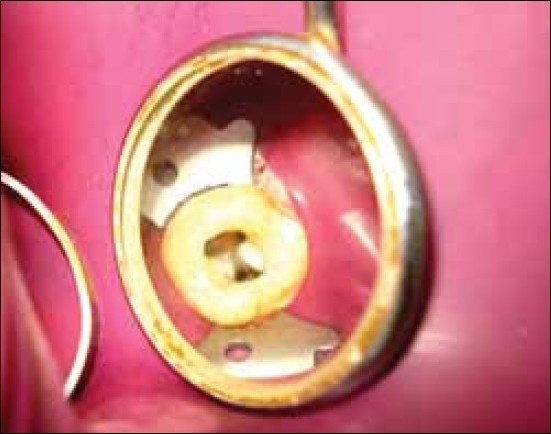
Occlusal view of the Access openening showing MP, DP, MB and DB canal orifices

**Figure 3:**
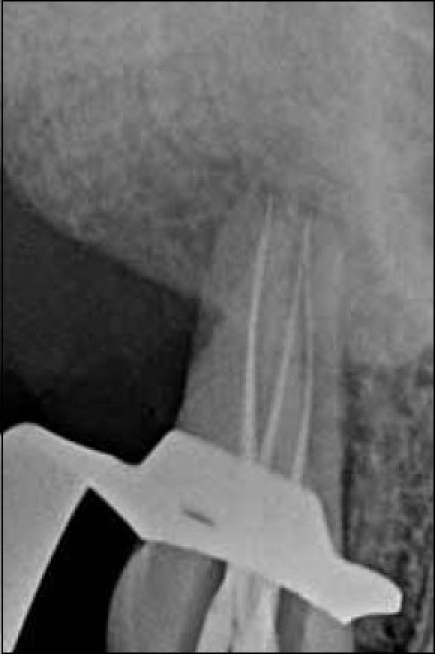
Working length radiograph showing files in all the four root canals

A diagnosis of chronic apical periodontitis was made for the tooth. Following local anesthesia, an endodontic access opening was made under isolation with Rubber Dam and the pulp chamber was exposed clearly. Initially conventional triangular access was prepared and three canals (MB, DB, P) were negotiated. As the palatal canal was located more distally {DP} and a hemorrhagic spot was visible towards the mesial side, with a groove joining both mesial and distal palatal canals, confirming two palatal canals [MP,DP] and access cavity was then modified to trapezoidal form. Examination of the chamber floor with an endodontic explorer (DG-16) revealed four canal orifices, mesiobuccal canal (MB), mesiopalatal canal (MP), distopalatal canal (DP) and distobuccal canal (DB) [Figures 2 and 3].

**Figure 4:**
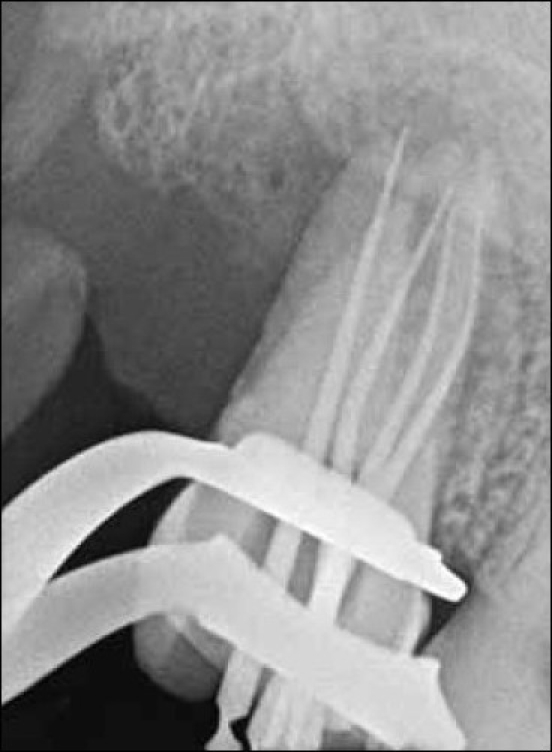
Chek in radiograph showing seated Master guttapercha cones in all the four canals

**Figure 5:**
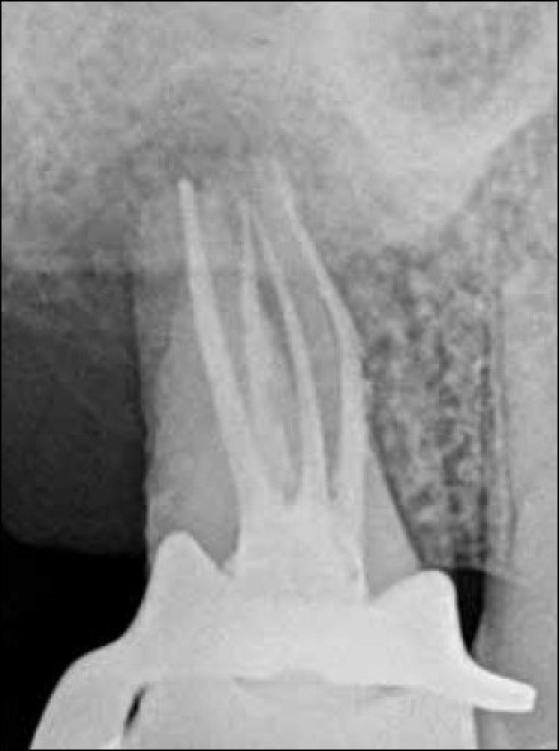
Post obturation radiograph

**Figure 6:**
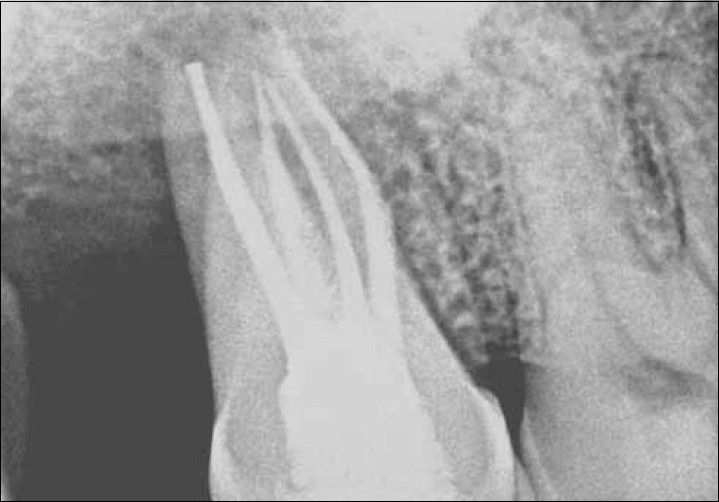
Six months followup radiograph

All canals were negotiated, patency was checked, and the working length was determined by using electronic apex locator Root ZX (J. Morita MFG. Corporation, Kyoto, Japan) and file in-radiograph with 15 no.K-files [Figures 4–6]. The root canals were cleaned and shaped by Rotary Ni- Ti Protaper System along with GLYDE (Dentsply Maillefer Company, USA) using crown down technique. The root canals were copiously irrigated with 2.5% sodium hypochlorite. Access cavity was temporized with TERM. Patient was recalled after one week; the tooth was asymptomatic. Root canals were irrigated again with normal saline and dried using paper points. Before obturation, master points were seated to test their suitability to canals and radiograph was taken.

The canals were obturated with selected master gutta-percha cone [variable taper] and AH-Plus sealer (Dentsply Maillefer Company, USA). The coronal gutta-percha cones were sheared off using heated instrument and vertical compaction was done using the heated pluggers at the individual canal orifices. A temporary restoration with IRM was placed. The patient was recalled after one week for post endodontic restoration and permanent adhesive composite restoration was done. At the six-month recall examination, the patient was comfortable without any symptoms.

## DISCUSSION

Maxillary second molar variants have already been reported in many clinical cases and *in vitro* studies. Peikoff *et al*,[6] carried out a retrospective study in 520 completed endodontic treatments of maxillary second molar teeth. They classified the anatomical root and canal variations into six categories: (1) Three separate roots and three separate canals; (2) three separate roots and four canals (two in the mesiobuccal root); (3) three roots and canals whose mesiobuccal and distobuccal canals combine to form a common buccal with a separate palatal; (4) two separate roots with a single canal in each; (5) one main root and canal; (6) four separate roots and four separate canals including two palatal. This study also revealed that occurrence of ‘standard’ configuration, i.e. three roots with three or four canals, was the most frequent (88.6%).

In addition to Peikoff’s results, a few less frequently occurred maxillary second molar variants have also been reported. Yang *et al*,[7] found that the maxillary second molars had a C-shaped root in 4.5% and C-shaped root canal orifices with C-shaped root canals in 4.9% in Chinese population. Some case reports presented four-rooted maxillary second molar with four separate canals in which three well-separated buccal roots were located either second distobuccal or mesiobuccal root.[8–10]

The present case report describes a new variant with four separate canals, mesiobuccal root, two individual palatal mesiopalatal and distopalatal with its own separate canal, and distobuccal root with a single canal. Compared with Peikoff’s categorization of the morphology of the root canal system, the variant identified in our case could be considered a combination of variant 2 and variant 6. Up to now, the variant with four separate roots and four separate canals including two palatal was the least frequent abnormality, with its incidence ranging from 1.47.to 2.1%.[11,12]

## CONCLUSION

The conservation of tooth structure must be kept in mind when establishing an endodontic access to allow for successful restoration of the tooth after root canal therapy.

Christie and Thompson[13] have recommended modifying the outline to an ovoid shape so that the roof of the chamber, when opened up, is more parallel to the mesial marginal ridge. They believe that this outline provides better access during the search for additional canals, therefore improving endodontic success. Maxillary second molars with two palatal canals often seem to have wider mesio-distal dimensions over the palatal cusps. Hence, a trapezoidal access opening would be more desirable than a triangular opening to identify two palatal canals to achieve success.
